# Clinical experience and complications with limited pleural dissection combined with a novel, simplified technique for thoracic Redon-like drain in vertebral body tethering

**DOI:** 10.1007/s43390-023-00760-4

**Published:** 2023-09-05

**Authors:** Aurélien Courvoisier, Marie-Christine Maximin, Olivier Daniel, Richard Gouron, Jean-Noël Evain, Alice Baroncini

**Affiliations:** 1 TIMC, University Grenoble Alpes, CNRS, UMR 5525, VetAgro Sup, Grenoble INP, CHU Grenoble Alpes, 38000 Grenoble, France; 2 Grenoble Alps Scoliosis and Spine Center, Grenoble Alps University Hospital, Bvd de la Chantourne, CEDEX 09, 38043 Grenoble, France; 3 Service d’Orthopédie Pédiatrique, CHU d’Amiens, Université Picardie Jules Verne, Chemin du Thil-CS 52501, 80025 Amiens Cedex 1, France; 4 Pediatric Anesthesia Department, Grenoble Alps University Hospital, Bvd de la Chantourne, CEDEX 09, 38043 Grenoble, France; 5 Department of Orthopaedics, RWTH Uniklinik Aachen, Pauwelsstraße 30, 52074 Aachen, Germany

**Keywords:** Vertebral body tethering, Chest drain, Suction bottle drain, Redon drain, VBT, Scoliosis

## Abstract

**Purpose:**

To report on our experience with a simplified, suction-bottle-drain technique of thoracic drain (Redon-like) combined with fully thoracoscopic vertebral body tethering (VBT) and a limited pleural approach, with particular focus on the rate of pulmonary complications.

**Methods:**

A retrospective study was performed on all consecutive patients who underwent VBT for adolescent idiopathic scoliosis. For all subjects, a 10G Redon drain, an active drain system consisting of a perforated tube and a suction bottle, was placed intrathoracically and tunneled under the skin. All drains were removed on the first postoperative day. Perioperative and postoperative data such as type of access, length of surgery, amount of fluid collection in the drain, and length of hospital stay were collected. The type and number of pulmonary complications occurring in the first 3 months after surgery, along with their symptoms and management, were recorded.

**Results:**

One Hundred eighty-two patients were included in the analysis. The mean length of surgery was 97 min (75–120). The average fluid collection in the drain was 30 ml (5–50), the mean length of hospital stay was 3 days (2–4). During the observation period, pulmonary complications occurred in five patients (2%). Two patients presented an aseptic right pleural effusion; for two patients, a residual pneumothorax was diagnosed on the X-rays in the recovery room and one patient developed a chylothorax. All patients recovered without sequelae.

**Conclusion:**

The simplified, Redon-like drain combined with a fully thoracoscopic VBT and limited pleural approach seems a safe and effective alternative to the chest drain. This technique allows to remove the drain on the first postoperative day, thus simplifying the management of the patients and improving their comfort.

## Introduction

Vertebral body tethering (VBT) is a recently developed surgical technique for the treatment of progressive and severe scoliosis. Surgery can be performed with different types of approaches, such as full thoracotomy, mini-thoracotomy with thoracoscopic assistance, and thoracoscopic only.

As VBT requires an intrathoracic access with lung deflation, the use of an intrathoracic drain is necessary to prevent the development of secondary pneumothorax and pleural effusion. Nonetheless, pulmonary complications are not infrequent and occur in 7–10% of patients [1–5]. While recent studies have showed that the short-term lung function is not affected by the development of these complications [6, 7], there is an ongoing effort toward identifying their possible risk factors [7]. Pulmonary complications may be related to many parameters: the length of the procedure, perioperative complications (rare), the type of approach, presence of a diaphragm split, and the type of drain used.

With the improvement of medical devices and technical skill, the time of VBT procedures has drastically decreased [8, 9]. However, there is still a degree of variability regarding the type of skin approach (mini open vs. thoracoscopic), pleural approach (one long incision vs. multiple small ones), and the type of used drain. In particular, a recent publication on 104 patients reviewed different chest drain systems in VBT, such as chest tubes and bulb drains: the drain was removed after 3 days on average, after accumulating 460 to 760 ml of fluids [10].

At our institution, we have been performing fully thoracoscopic VBT with a limited pleural approach, namely small pleural incision about 3 mm long with coagulation of the segmental vessels in the middle of the lateral aspect of each vertebral body, rather than a craniocaudal incision over all the instrumented vertebrae. Furthermore, we have been employing a simplified technique of thoracic drain, namely a tunneled Redon drain, as described in 1998 by Ruzic et al. for anterior scoliosis surgeries [11]. This closed drain technique consists of a rigid, perforated tube connected to a suction bottle that presents numerous advantages in comparison to the more commonly used chest drain, and the drain can be removed on the first postoperative day. The smaller size of the tube and drain system allows for a less painful removal and for an increased comfort of the patients. Furthermore, suction bottle drains are easier to handle at the ward and may represent a more cost-effective option for institutions that require patients to be monitored at the intensive care unit as long as the chest drain is in place [8]. Lastly, the Redon skin incision does not need to be sutured, while the chest drain skin incision needs to be sutured with a purse-string stitch upon removal, which can result in an aesthetically unpleasant scar.

The purpose of the study was to report on our 10-year experience with a limited pleural approach and a Redon-like intrathoracic drain, to illustrate the used technique and analyze the rate and type of pulmonary complications.

## Materials and methods

This retrospective, two-centre study was conducted in accordance with the Declaration of Helsinki, with the current regulation and reference methodology MR-004 and with the STROBE statement [12]. The study was approved by the SFAR Ethical Committee (IRB 00010254-2021-202).

### Patient population

All consecutive patients who underwent VBT for adolescent idiopathic scoliosis (AIS) between July 2018 and September 2022, and who had a minimum follow-up of 3 months, were screened for inclusion.

Surgical revisions and patients who were opposed to data collection were excluded from the analysis.

### Surgical technique and drain placement

The detailed surgical technique was described in a previous publication [8].

**Fig. 1:**
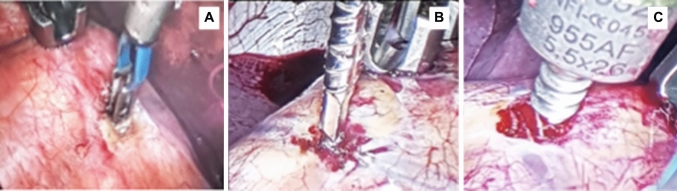
The following video screenshots show the three main stages of the pleural approach and screw placement. **A** Segmental vessels coagulation. **B** Tapping with the threaded electronic conductivity device. **C** Screw placement

In sum, thoracic procedures were performed with a fully thoracoscopic approach. Surgery was performed through a fully thoracoscopic approach with one-lung ventilation in left lateral decubitus. Four to five incisions on the mid-axillary line (15 mm—trocar size) were typically sufficient to conduct the entire procedure. The approach to the vertebral pleura consisted in a small coagulation of the segmental vessels (about 2–3 mm) in the middle of the lateral aspect of each vertebral body (Fig. 1A), as opposed to a long craniocaudal incision over the instrumented vertebrae. The tap trajectory was secured with a threaded electronic conductivity device to limit radiation exposure (Fig. 1B) [13]. The screws were then inserted in the vertebral body (Fig. 1C).

**Fig. 2:**
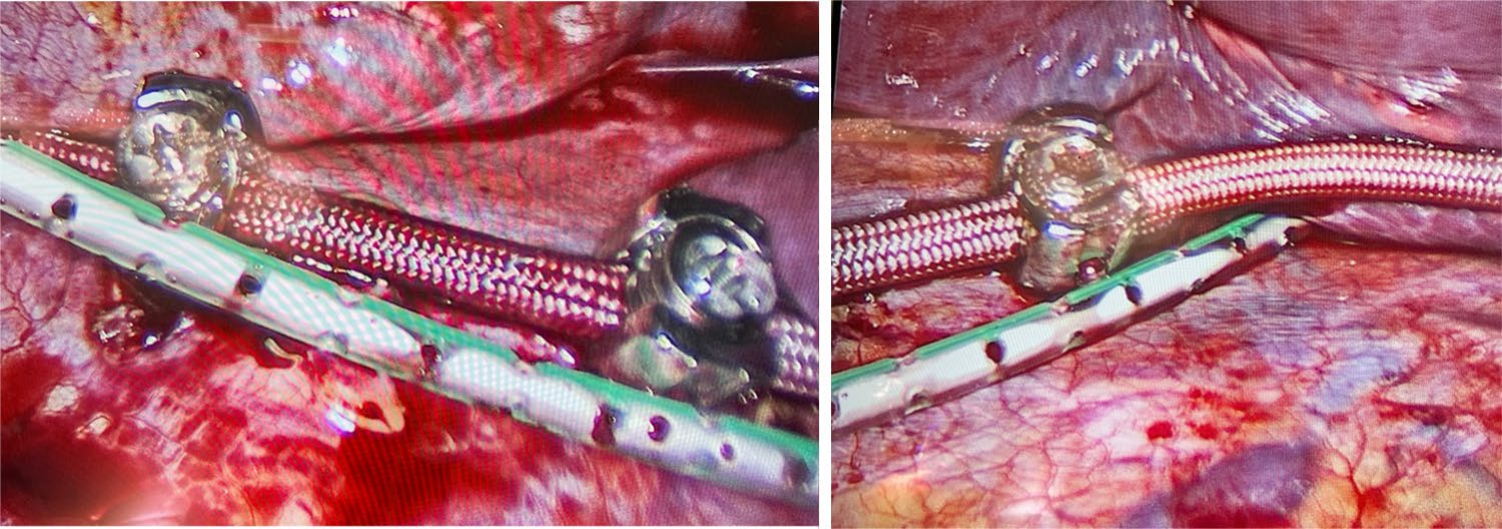
Video screen captures showing the drain position alongside the screws and tether

**Fig. 3:**
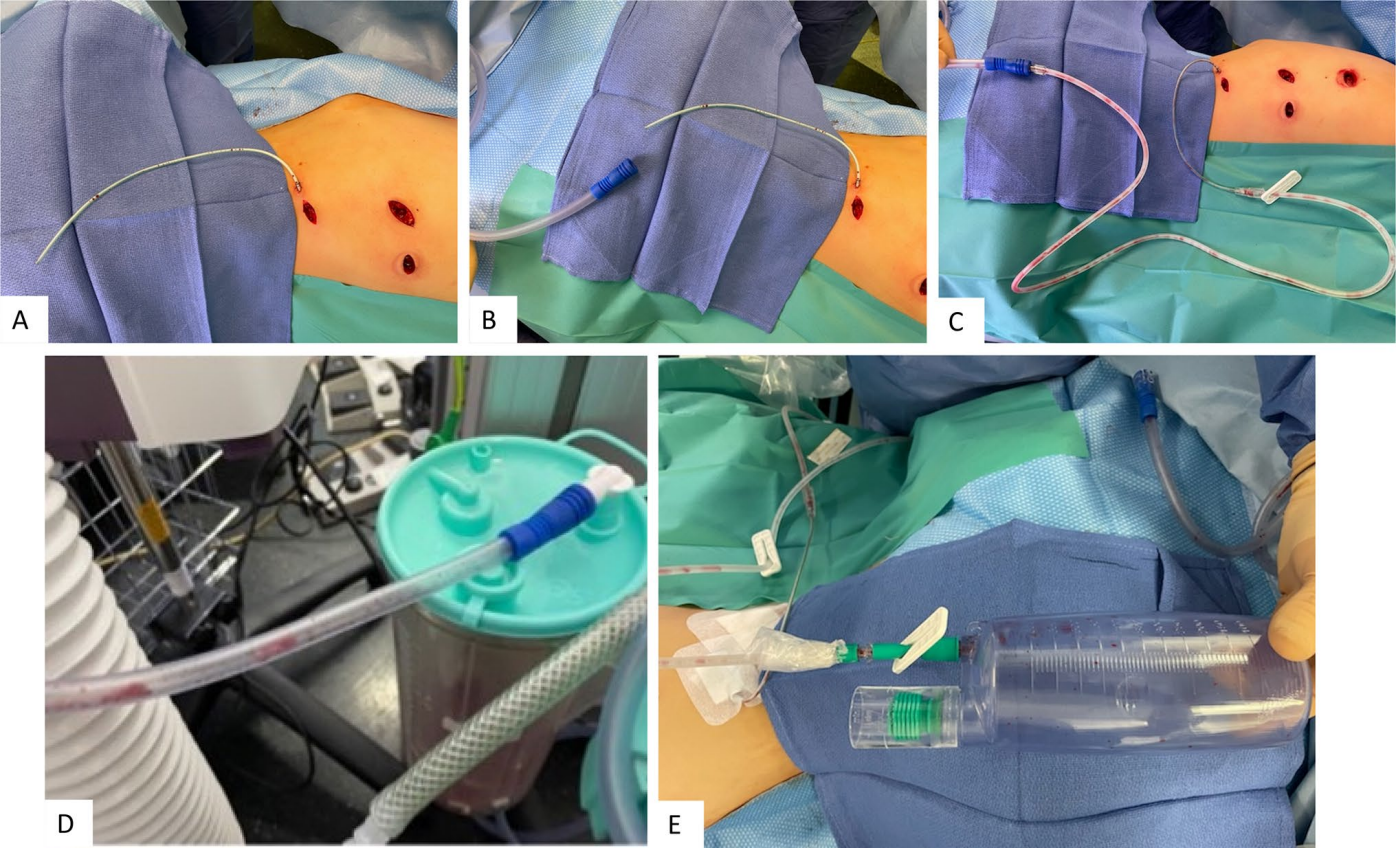
Photographs showing the management of the drain during wound closure. The tube was tunneled and secured to the skin (**A**), and then connected to the room suction system until the skin was closed (**B**–**D**), to eliminate any residual fluids and pneumothorax. After skin closure, the drain was disconnected from the room suction system and connected to the negative pressure bottle, which allowed to drain any residual fluids from the chest (**E**). Of note, in picture E, the bottle still held the void, indicating the absence of residual pneumothorax

At the end of the procedure, a 10G Redon drain was placed alongside the screws and tether, with the tip of the drain positioned next to the most cranial screw (Fig. 2). This active, closed drain system consists of a rigid, perforated tube that is connected to a suction bottle. The tube was tunneled under the skin and secured (Fig. 3A). During wound closure, the drain was connected to the suction system of the room. At the same time, the anesthesiologist re-inflated the lung. The combination of both procedures helped remove the residual pneumothorax. Once the skin was closed, the tube was connected to the Redon drain which held the void (Fig. 3B–D).

In case of lumbar procedures, a trans-thoracic approach was used for T11-L1 and a mini-lumbotomy with a trans-psoas approach was performed to instrument the vertebrae caudal to L2. A small incision was performed in the diaphragm pillar close to the spine to slide the tether from the thorax down to the lower screws. One Redon drain was placed intrathoracically as described above, and one in the mini-lumbotomy access.

### Postoperative management

A chest X-ray was performed in the recovery room to ensure that there was no residual pneumothorax. The patients were then moved to the ward.

The drain was removed systematically on day 1 in the morning, irrespectively of the amount of collected fluid [11]. Pulmonary physiotherapy was initiated on day 1 under the guidance of a physiotherapist and with the aid of a tri-flow for 5 to 10 min, 4 to 5 times a day [7, 14]. While this manuscript focusses on primary cases, the same drain system and postoperative management have been used in revision cases as well.

While serial chest X-rays were not performed in patients without pulmonary complications, the full-spine EOS scans performed on the second postoperative day allowed to rule out residual pneumothorax or pleural effusion.

All i.v. lines were removed on the first postoperative day and substituted with oral medication. The patients usually stood up and walked on day 1 and were discharged 2 or 3 days after surgery.

All patients were required to wear a brace for 6 weeks after surgery. The pulmonary physiotherapy was continued at home for 2 weeks. Noncontact sports (swimming, biking) were authorized 6 weeks after surgery. Full return to sport was authorized after 3 months, with the exception of collision sports such as rugby which were authorized after 12 months.

In case of pulmonary complications, daily X-rays were taken until the complication was resolved.

### Outcomes of interest

Baseline demographic data such as gender, age at surgery, and Lenke curve type were collected. The type of approach (thoracic, lumbar, bilateral), the level of the lowest instrumented vertebra (LIV), and the length of surgery were also recorded.

Postoperative data such as the amount of fluids collected in the drain, the requirement of monitoring at the intensive care unit, and the length of the hospital stay were collected.

The rate of the surgical-approach-related complications (e.g., vascular or organ damage with trocars, screws or other instruments) and the rate of pulmonary complications were evaluated at the 3-month follow-up. For each recorded complication, the timing, onset symptoms, and management were recorded.

## Results

### Patient selection and demographic data

**Fig. 4:**
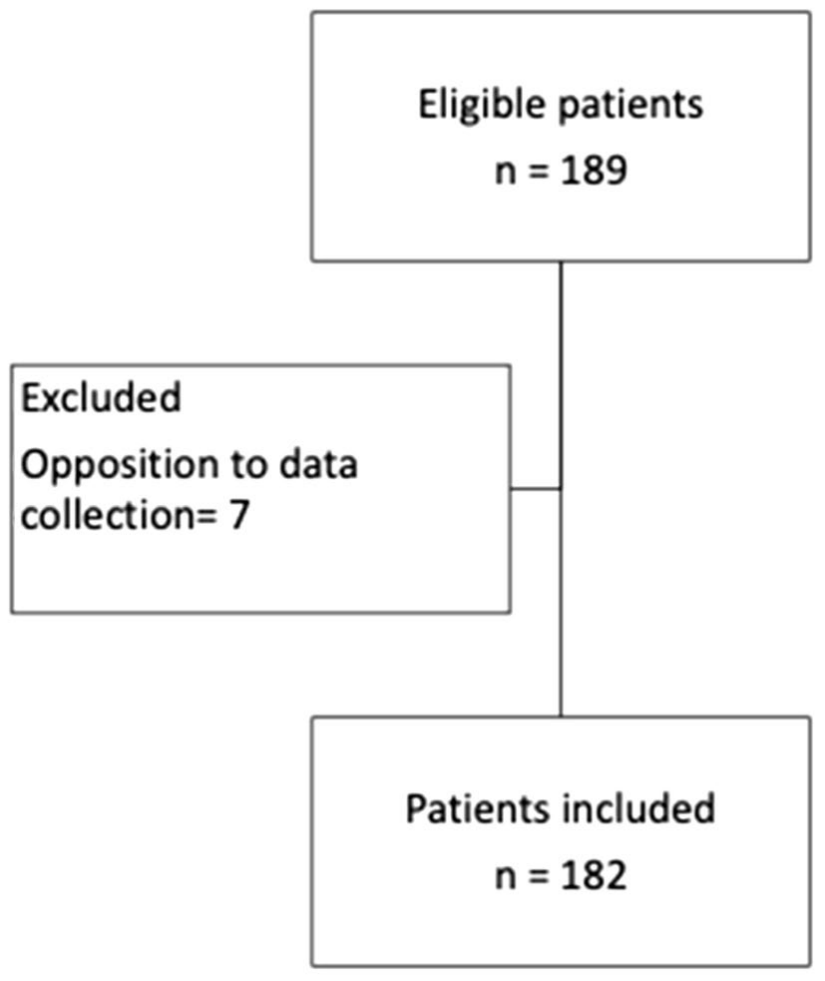
Flowchart of the patient selection process of the study

During the observation period, 189 patients who met the inclusion criteria were screened at the 2 participating sites. Seven patients were excluded because they were opposed to data collection, so that data from one hundred eighty-two subjects were available for this analysis (Fig. 4).

The baseline and perioperative characteristics of the patients in the series are described in Table 1.

**Table 1:** Summary of the preoperative and perioperative characteristics of the patients

Patient characteristics	*N*
Age surgery (years)	12.5 (9–16)
Preoperative Cobb angle (°)	49 (40–58)
Postoperative Cobb angle (°)	27 (15–39)
Follow-up	13 months (3 months—7 years)
Gender	
F	152 (84%)
M	30 (16%)
Lenke type	
1A	76 (46%)
1B	29 (16%)
1C	36 (20%)
5C	28 (16%)
2A	14 (8%)
Approach	
Single thoracic	135 (74%)
Single lumbar	28 (16%)
Bilateral	19 (10%)
Right thoracic LIV	
T11	13 (9%)
T12	96 (62%)
L1	42 (27%)
L2	3 (2%)

Data are expressed as mean (range) for continuous data and as number (percentage) for proportions. Of note, T11 was chosen as the LIV only in case of bilateral procedures

### Outcomes of interest

The length of surgery was 97 min in average (range 75–120). On the first postoperative day, the amount of fluid collected in the drain was 30 ml (range 5–50). None of the patients required postoperative monitoring at the intensive care unit and the mean length of hospital stay was 3 days (range 2–4).

During the observation period, pulmonary complications were observed in five patients (2%). For all other patients, residual pneumothorax or pleural effusion were ruled out at the routinary 3-month follow-up EOS scans performed to evaluate curve correction and screw placement. Two of the five patients with complications presented an aseptic right pleural effusion diagnosed because of chest pain, coughing and breathing difficulties, both at 6 weeks post-op. Both patients required re-hospitalization and the insertion of a traditional chest drain, and fully recovered in 5 days. The chest drain was set on continuous suction and was removed once the output was < 20 ml/24 h. For both patients, daily X-rays were taken to monitor the pleural effusion and once after removal of the chest drain.

For two further patients, a residual pneumothorax was diagnosed on the X-rays in the recovery room. Along with the standard care with Redon drain and pulmonary physiotherapy, both patients required oxygen therapy. The pneumothorax resolved in 2 days without the need for a more invasive drain. Both patients underwent daily X-rays controls until the radiologic findings of pneumothorax resolved.

One last patient presented a chyle collection in the drain on the first postoperative day. A fat-free regimen was started and subsequently, a lymphography was performed and showed a chyle leak close to the screw at T11 level on the left side. The interventional radiologist attempted a CT-guided embolization during the lymphography, but this could not be performed as the vessel was too small. Thus, during the same intervention, a CT-guided sclerosis of the leakage was performed instead, and the patient recovered without sequelae. The drain that was placed intraoperatively was not substituted with a traditional chest drain and was kept in place for 2 weeks. In this case, daily X-rays were performed for the first week and later weekly until complete recovery.

For all five patients, the pulmonary function test performed 1 year postoperatively did not show any reduction in pulmonary function in comparison to the expected values for healthy, same-age subjects. A summary of the observed pulmonary complications is presented in Table 2.

**Table 2:** Summary of data regarding the observed pulmonary complications

Complication	N. of patients	Curve type	Treatment	Time to diagnosis
Aseptic pleural effusion	2 (1%)	Right thoracic	Drainage 2 weeks	45 days po
Pneumothorax	2 (1%)	Right thoracic	Drainage 2 days	Immediate post-op
Chylothorax	1 (0.5%)	Double	Fat-free diet plus sclerosis	Day 1 po

No other complications to vessel or organs were recorded.

## Discussion

The main finding of this study was that the presented drain technique, associated with a full-thoracoscopic surgical technique and minimal pleural invasiveness, allowed a rapid removal of the drain and was associated with a low rate of pulmonary complications (2%).

According to the data available in the literature, pulmonary complications after VBT are not uncommon and have been observed in 7–10% of patients [1–3, 7]. Commonly reported complications in the literature were recurrent pulmonary effusion, chylothorax, atelectasis, pneumonia, and pneumothorax [2]. In the presented series, the rate of observed pulmonary complications was much lower than the reported rate in the literature, but the kind of observed complications was similar.

As a routine, most authors use a chest drain after surgery to avoid pneumothorax and pleural effusion [15]. However, there is no consensus as to how long the drain is kept in place: some authors remove it when the output in 24 h is below 100 ml [7], while others wait until the output is below 20 ml [9]. This, of course, reflects on the time for which the drain stays in place, which has been reported to average 2–3 days but has been prolonged to up to 5 days [9].

While leaving the drain for a prolonged time ensures a detailed monitor on the amount and quality of the output (e.g., bloody vs. serous vs. chyle), the chest drain, typically thicker than a Redon drain, might irritate the pleura, and thus cause itself an increase of the output. Of note, the amount of fluids collected in the drain in the presented cohort was almost 20 times lower than the amount recorded with the chest or bulb drains [10]. Since the Redon drain was always removed on the first postoperative day, the number of days for which the drain was in place was also considerably lower in the presented cohort in comparison to the data presented by Haber et al. for chest and bulb drains (2 to 3 days) [10]. Furthermore, the presence of the drain bottles limits the mobility of the patients. For these reasons, we adopted Ruzic’s technique [11]: the use of a thinner Redon drain potentially limits pleural irritation and removing the drain early allows the patient to mobilize more easily and with less pain.

A further advantage of using a Redon drain is that some institutions require patients to be in the intensive care unit until the chest drain is removed [8]. The use of a type of drain that is easy to handle at the ward, and with which all nurses are familiar, might reduce the need of intensive care monitoring and, in turn, reduce the costs of postoperative care. The use of a Redon-like drain that can be removed on the first postoperative day can also improve the patients’ comfort and allows them to mobilize quickly and with ease. Also, as the drain is thin and is tunneled under the skin, the skin incision does not require to be sutured. This represents a further advantage of the Redon against the chest drain, which usually requires a purse-string stitch that can leave a conspicuous scar—an aspect that is even more relevant considering the young age of the patients who undergo VBT.

The question raises, whether the difference in pulmonary complication rate between the presented cohort and data available in the literature could be caused by other factors rather than the type of drain and access alone. Comparing the baseline data of the present cohort with those obtained from the literature, age at surgery was not dissimilar (11.7 vs. 12.5 years) and in both groups, the majority of patients presented thoracic curves [2]. Thus, it is unlikely that the observed lower rate of pulmonary complications is due to a difference in the patients’ characteristics. Trobisch et al. have reported a higher incidence of pulmonary complications when crossing the diaphragm or choosing LIV at T11 [7]. Both instances are a rare situation for short right thoracic curves, where the LIV is usually on T12 or L1. Placing L1 screws often requires releasing the crus of the diaphragm from the vertebral body and pushing it downward. We did not encounter a higher incidence of pulmonary complication in those cases; however, the limited number of observed complications does not allow to perform an analysis of possible risk factors. In the presented cohort, L2 was the LIV for only three patients and L2 placement was performed through thoracoscopy. This procedure requires a wider dissection of the diaphragm to secure L2 placement. In these three cases, no complication was reported. Crossing the diaphragm is usually necessary when performing VBT on lumbar curves. Our technique combines a thoracoscopic approach screws upper than L2 and a lumbotomy for L2, L3, and L4 screws. A small hole in the diaphragm close to the L1 screw is used to slide the tether from the thorax down to the lumbotomy. In those cases, we did not encounter any specific pulmonary complications. The left chylothorax reported in the study was due to a leak close to the left T11 screw: while this finding supports the hypothesis that T11 LIV might represent a risk factor for pulmonary complications, further studies will be required to reach a definitive conclusion.

Ruzic et al. were the first to publish a series reporting the use of the described drain technique for complex anterior surgeries for pediatric deformities, recommending the use of a Redon drain when a hermetic closure of the pleura was possible and there was no air leakage present [11]. The authors found that the use of a Redon drain did not raise the rate of complications in comparison to a standard chest drain [11]. As VBT allows to respect soft tissue, bone, and lungs, and the screw heads seal the defect in the pleura, this technique perfectly fits the recommendation given by Ruzic et al. for the use of Redon chest drain after anterior scoliosis surgery.

This study does not come without limitations, the main one being its retrospective nature. The low number of observed complications does not allow to perform an analysis of the risk factors that might be associated with pulmonary complications: further studies on a much larger cohort will be required to clarify this point. Furthermore, as the presented approach and drain techniques have been employed at the involved institutions since the beginning of the experience with VBT, a direct comparison with different approaches or drain systems could not be performed.

## Conclusion

The simplified chest drain technique with a tunneled Redon-like drain, combined with a full-thoracoscopic procedure and limited pleural approach, seems to offer a safe and effective alternative for AIS patients undergoing VBT. This option allows to remove the drain on the first postoperative day, and might, thus, provide a more comfortable and cost-effective alternative to the more widely used chest drain.

## Data Availability

Data can be made available in anonymized form upon reasonable request.
